# The prevalence of disability and associated factors among community adults in the baseline of CHCN-BTH Cohort Study

**DOI:** 10.1186/s12889-023-15066-3

**Published:** 2023-09-05

**Authors:** Hui-Ping Zhu, Han Qi, Xiao-Hui Liu, Kuo Liu, Bing-Xiao Li, Fu-Yuan Wen, Yun-Yi Xie, Ling Zhang

**Affiliations:** 1 Department of Epidemiology and Health Statistics, School of Public Health, Capital Medical University, and Beijing Municipal Key Laboratory of Clinical Epidemiology, Beijing, 100069 China; 2 Beijing Key Laboratory of Mental Disorders, National Clinical Research Center for Mental Disorders & National Center for Mental Disorders, Beijing Anding Hospital, Capital Medical University, Beijing, 100088 China

**Keywords:** Disability, Prevalence, Chronic physical conditions, CHCN-BTH, Multilevel logistic regression

## Abstract

**Background:**

Disability was a major public health problem in China. However, the prevalence of disabilities in community-dwelling adults and their relationships to chronic physical conditions were unclear. We aimed to estimate the prevalence of disabilities and associated factors among a large community-based cohort in China.

**Methods:**

Participants who were local permanent residents aged 18 years or above and completed the disability assessments were selected from the Cohort study on Chronic Disease of Communities Natural Population in Beijing, Tianjin and Hebei (CHCN-BTH) from 2017 to 2019. Disability was assessed using five questions about impairments and activity limitations based on the International Classification of Functioning (ICF), Disability and Health. Univariate, multivariate and multilevel logistic regressions were conducted to estimate the associations between disabilities and associated factors.

**Results:**

Totally, 12,871 community-dwelling adults completed the survey. Among of them, 12.9% (95% CI: 12.3%-13.5%) reported having any disability. The prevalence of any disability was significantly higher in participants who were older age, widowed, retired and smokers, had higher BMI, average monthly income < 5000 RMB, lower education level, lower physical exercise frequency and heavy physical labor. Multilevel logistic regressions showed that there were significant associations between disabilities with chronic physical conditions, especially in the vision impairment with lower back pain, and hearing impairment as well as difficulty walking without special equipment with injuries.

**Conclusions:**

Many Chinese adults suffered from disabilities. Sustained efforts should be made to develop specific population-based health promotion and prevention programs for disabilities in China.

**Trail registration:**

ChiCTR1900024725 (25/07/2019).

**Supplementary Information:**

The online version contains supplementary material available at 10.1186/s12889-023-15066-3.

## Introduction

Over a billion people globally live with some form of disability, and the majority living in low- and middle-income countries (LMICs) [1, 2]. People with disability may experience greater vulnerability to co-morbid conditions, secondary conditions, and engaging in health risk behaviors [3]. According to the Second National Disability Survey in China, it was estimated that 84.6 million people had a disability in 2006. Of them, more than 44.0 million were aged 60 years and over [4, 5]. The precise prevention and identification of people with disability are of great significant to relief the burdens of society and family.

The definitions of disability varied from “body structure impairment”, “capacity limitations” to “participation restriction” with the development of model medicine patterns [6, 7]. To increase the comparability of disability across nations, WHO published a classification system of disability called “The International Classification of Functioning, Disability and Health (ICF)”, and defined disability as “an umbrella term for an impairment of body structure, a limitation of activity or a restriction in participation, and the interaction between individuals with a health condition and personal and environmental factors” [8]. Several assessment tools for disability had been developed based on the elements of ICF, such as the WHO disability assessment schedule second edition (WHODAS 2.0) [9], the Washington group short set of questions on disability (WGSS), as well as the six questions about disability status that develop by the Washington Group and had been incorporated by the U.S National Health Interview Survey (NHIS) in the population and housing censuses from 2016 to 2020 [10–12].

Although ICF gained wide acceptance and had more domains that can provide more comprehensive information about the disability, its operational measurement varies greatly among studies. Low income and developing countries tend to adopt a measure focused on a narrow definition of impairments and report a lower disability prevalence rate than high-income countries because of the poor economics, weak social security system and some other sociocultural factors [13, 14]. In China, two large-scale nationally representative household surveys were conducted in 1987 and 2006, and the prevalence of disability was 4.9% and 6.5%, respectively [4, 5]. Lestari et al. used a cross-sectional data from the WHO longitudinal multi-country Study on Global AGEing and Adult Health (SAGE) Wave 1 (2007–2010), and identified the prevalence of disability in Chinese people aged 50 years and over was 16.2% [15]. The 2006 national survey took into account the ICF, but the evaluations of disabilities were mostly relied on the physiological impairment rather than the functional barriers and social adaptability [16]. In the China Health and Retirement Longitudinal Survey (CHARLS), the disabilities were evaluated with the self-reported physical impairments and limitations in activities of daily living (ADL) and instrumental activities of daily living (IADL). The prevalence of disability would also be underestimated due to unistructural measurement [17]. It is necessary to investigate the disability using ICF-based approach to improve the comparability across countries.

China is facing an unprecedented pace of aging that will contribute to disability levels, and the burden of disability is predicted to increase as a consequence of population ageing [15]. Knowledge about the prevalence and associated factors of disability is important for the development of specific population-based health promotion and prevention programs. However, more recent estimates about disability that based on the ICF framework were not available, especially estimates gained from a large representative sample of the common population. Also, few studies explored the association between sociodemographic factors, health behaviors, and chronic condition with disability in China. Therefore, we aimed to utilize the ICF-based approach to identify the prevalence and factors associated with disability among a representative sample of community-dwelling general population in Northern China.

## Methods

### Data source and participants

We obtained data from the baseline survey from 2017 to 2019 of the Cohort Study on Chronic Disease of Communities Natural Population in Beijing, Tianjin, and Hebei (CHCN-BTH), which was registered with the Chinese Clinical Trial Registry, number ChiCTR1900024725. CHCN-BTH was one of the large national prospective cohorts in China and aimed to monitor the prevalence of major non-communicable chronic diseases (NCDs) and their epidemic trends, and to demonstrate the etiology and pathogenesis of chronic diseases from the aspects of genetic, environmental and lifestyle aspects. A multistage, stratified cluster sampling method was used to derive a representative sample of the community-dwelling population in Beijing-Tianjin-Hebei region based on administrative areas, local air pollution exposure characteristics and local gross domestic product. More detailed information on study design and methods have been published elsewhere [18].

Eligible participants who were local permanent residents, aged 18 years or above, and completed the disability survey at baseline stage were recruited in this study. A total of 15,219 individuals living in Beijing and Tianjin region completed the disability surveys and 2348 participants were excluded due to missing data of key covariates. Finally, 12,871 (85.0%) participants were included in this analysis. The statistical power analysis of “differences between proportions” showed that the power was greater than 0.9, indicating that the sample size of 12,871 was large enough (alpha = 0.05, null proportion = 0.1, binomial proportion = 0.129, method = exact). Survey questionnaire was reviewed by leading national and international experts. Respondents were interviewed face-to-face by trained student interviewers from Capital Medical University and Peking Union Medical College, and medical staff interviewers who worked in local community healthcare service where the interviews took place. The study was approved by the Institutional Review Board of the Center of Disease Control and Capital Medical University. Informed consent form was obtained in writing from all participants at their enrollment.

### Covariables

Sociodemographic and behavioral variables were regarded as covariables in this study. Sociodemographic variables included age group (18–29, 30–39, 40–49, 50–59, ≥ 60 years old), gender (men, women), average monthly income of each family member (< 5000 RMB, ≥ 5000 RMB), education level (elementary school and below, middle or high school, college and above), marital status (unmarried, married, divorced, widowed), occupation (worker, professional, retirement, others). Body mass index (BMI) was a person’s weight in kilograms divided by the square of height in meters. In this study, BMI was categorized as < 24.0 kg/m^2^, 24.0–27.9 kg/m^2^, ≥ 28.0 kg/m^2^ [19]. Furthermore, physical labor (mild: such as sitting at a desk all day at work, or walking less during work days, moderate: such as assembly line work, driving, electrical installation, heavy: carrying heavy items, lumbering, mining, dancing), physical exercise (5–7 days per week, 3–4 days per week, 1–2 days per week, ≤ 3 days per month, never), smoking (nonsmoker, smoker) and drinking (nondrinker, drinker) were behavioral variables. Physical exercise was defined as regular activities for the purpose of health, such as running, swimming, cycling, climbing, dancing, and walking, which should last 30 min or longer a day [20].

### Chronic physical conditions

In this study, several common chronic physical conditions including hypertension, diabetes, dyslipidemia, coronary heart disease and stroke, chronic bronchitis (CB) and chronic obstructive pulmonary disease (COPD), digestive diseases (peptic ulcer, gastritis, gallstones or cholecystitis), benign and malignant tumors, asthma, injuries (fracture, accidental injury) or lower back pain were incorporated to explore their associations with disabilities [21]. Previous diagnoses from secondary or tertiary hospital were needed to confirm the chronic physical conditions. The details of the investigations about chronic physical conditions were introduced elsewhere [20].

### Disability definition

Disability was assessed using five questions about impairments and activity limitations which is in line with the previous study conducted by Alhajj et al. [22]. These questions adopted and modified survey questions of the US. National Health Interview Survey (NHIS) about disabilities and were conceptually based on the ICF [12, 23]. The last two questions reflect limitations in performing activities of daily living (ADL) and instrumental activities of daily living (IADL). Individuals who answered “yes” to at least one of the following questions were identified as having any disabilities:

1. Vision impairment: Are you blind or do you have serious difficulty seeing, even when wearing glasses?
2. Hearing impairment: Are you deaf or do you have serious difficulty hearing?
3. Difficulty walking without special equipment: Because of a health problem, do you have serious difficulty walking (need special equipment)?
4. ADL: Because of a physical, mental, or emotional problems, do you need the help of other persons with personal care needs, such as eating, dressing, bathing, or getting around inside the home?
5. IADL: Because of a physical, mental, or emotional problems, do you need the help of other persons in handling routine needs, such as everyday household chores, doing necessary business, shopping, or getting around for other purposes?

### Statistical analysis

All analyses were performed using SAS 9.4 statistical software (SAS institute Inc., SAS Campus Drive, Cary, NC, USA). Continuous variables were described using mean ± standard deviation, and categorical variables were n (%). The prevalence of disability by covariables and chronic diseases were calculated and compared using Chi-square tests or the Fisher exact probability. Multivariate and multilevel logistic regression models were constructed to examine the associations between chronic physical conditions with disability. Odds ratios (ORs) and their 95% confidence intervals (CIs) were estimated from these models. Akaike information criterion (AIC) was used to reflect the goodness of model fitting. Specifically, two types of logistic models were built for each chronic physical condition: (1) multivariate logistic analysis adjusted for sociodemographic variables (age group, sex, education level, occupation, marital status, average monthly income of each family member, BMI) and behavioral variables (physical labor, physical activities, drinking, smoking); (2) multilevel logistic analysis that regarded subjects as level 1 and regions (Beijing or Tianjin) as level 2 adjusting for the same variables as model 1. The PROC GLIMMIX and NLMIXED in SAS were used for the multilevel logistic regressions. A *P* value less than 0.05 was considered statistical significance.

## Results

### Characteristics of participants

Totally, 12,871 individuals aged 18 years or older were included in the analysis. Of them, 54.1% were men (*n* = 6960), and the average age was 48.57 ± 15.20 years old. The unadjusted prevalence of any disability in the present sample was 12.9% (95% CI: 12.3%-13.5%) in general. The unadjusted prevalence of disability was higher in Tianjin (15.0%, 95% C: 14.1%-15.8%) than Beijing (10.7%, 95% CI: 9.9%-11.5%). The unadjusted prevalence of hearing impairment, vision impairment, difficulty walking without special equipment, ADL and IADL disability were 7.1 (95% CI: 6.6%-7.5%), 5.1 (95% CI: 4.7%-5.5%), 2.3% (95% CI: 2.0%-2.6%), 2.5% (95% CI: 2.2%-2.8%) and 2.3% (95% CI: 2.0%-2.5%), respectively.

### Univariate analysis of the prevalence of disabilities with sociodemographic characteristics, behavioral factors, and chronic physical conditions

The unadjusted prevalence of any disabilities by various variables were presented in Table 1. The prevalence of any disabilities was significantly higher in participants who were older age, widowed, retired, smokers, lived in Tianjin province, had higher BMI, having average monthly income < 5000 RMB, had lower education level, had lower physical exercise frequency, and had heavy physical labor. Of them, participants from Tianjin had significant older average age (55.44 ± 12.49 vs 41.03 ± 14.32 years old), higher percentage of women (62.4% vs 27.9%), higher proportion of obesity (27.1% vs 15.9%), lower educational level (proportion of college and above: 19.4% vs 78.6%) and lower income level (proportion of monthly individual’s income ≥ 5000 RMB: 7.8% vs 57.8%) than Beijing.

**Table 1:** Unadjusted prevalence of any disabilities by sociodemographic characteristics and behaviors among community-dwelling individuals in CHCN-BTH study (*n* = 12,871)

Variables	Total	With any disabilities		*P* value
	**N (%)**	**n**	**% (95% CI)**	
**Sociodemographic variables**				
**Age group (y)**				< 0.001
18–29	1652	78	4.7 (3.8–5.8)	
30–39	2563	165	6.4 (5.5–7.4)	
40–49	2287	254	11.1 (9.9–12.4)	
50–59	3113	480	15.4 (14.2–16.7)	
≥ 60	3256	687	21.1 (19.7–22.5)	
**Sex**				0.555
Men	6960	911	13.1 (12.3–13.9)	
Women	5911	753	12.7 (11.9–13.6)	
**Region**				< 0.001
Beijing	6135	656	10.7 (9.9–11.5)	
Tianjin	6736	1008	15.0 (14.1–15.8)	
**BMI (kg/m**^**2**^**)**				0.004
< 24	4457	521	11.7 (10.8–12.7)	
24–28	5612	744	13.3 (12.4–14.2)	
≥ 28	2802	399	14.2 (13.0–15.6)	
**The average monthly income of each family member (RMB)**				< 0.001
< 5000	8801	1269	14.4 (13.7–15.2)	
≥ 5000	4070	395	9.7 (8.8–10.6)	
**Marital status**				< 0.001
Unmarried	1403	73	5.2 (4.1–6.5)	
Married	10,680	1416	13.3 (12.6–13.9)	
Divorced	312	55	17.6 (13.7–22.1)	
Widowed	476	120	25.2 (21.5–29.3)	
**Education**				< 0.001
Elementary school and below	1098	197	17.9 (15.8–20.3)	
Middle or high school	5641	853	15.1 (14.2–16.1)	
College and above	6132	614	10.0 (9.3–10.8)	
**Occupation**				< 0.001
Worker	2667	355	13.3 (12.1–14.6)	
Professional^a^	2226	134	6.0 (5.1–7.1)	
Retirement	2071	320	15.5 (13.9–17.1)	
Others^b^	5907	855	14.5 (13.6–15.4)	
**Health behavioral variables**				
**Physical labor**				< 0.001
Mild	9647	1256	13.0 (12.4–13.7)	
Moderate	2717	317	11.7 (10.5–12.9)	
Heavy	507	91	17.9 (14.8–21.5)	
**Exercise frequency**				< 0.001
5–7 days per week	4298	638	14.8 (13.8–15.9)	
3–4 days per week	1785	184	10.3 (9.0–11.8)	
1–2 days per week	2566	228	8.9 (7.8–10.0)	
≤ 3 days per month	1340	132	9.9 (8.3–11.5)	
Never	2882	482	16.7 (15.4–18.1)	
**Smoking status**				< 0.001
Nonsmoker	8459	994	11.8 (11.1–12.5)	
Smoker	4412	670	15.2 (14.1–16.3)	
**Drinking status**				0.640
Nondrinker	7339	940	12.8 (12.1–13.6)	
Drinker	5532	724	13.1 (12.2–14.0)	

*BMI* Body mass index; ^a^Professional includes doctors, nurses, teachers, engineers, accountants, lawyers; ^b^Others includes farmers, students, salespeople

**Fig. 1:**
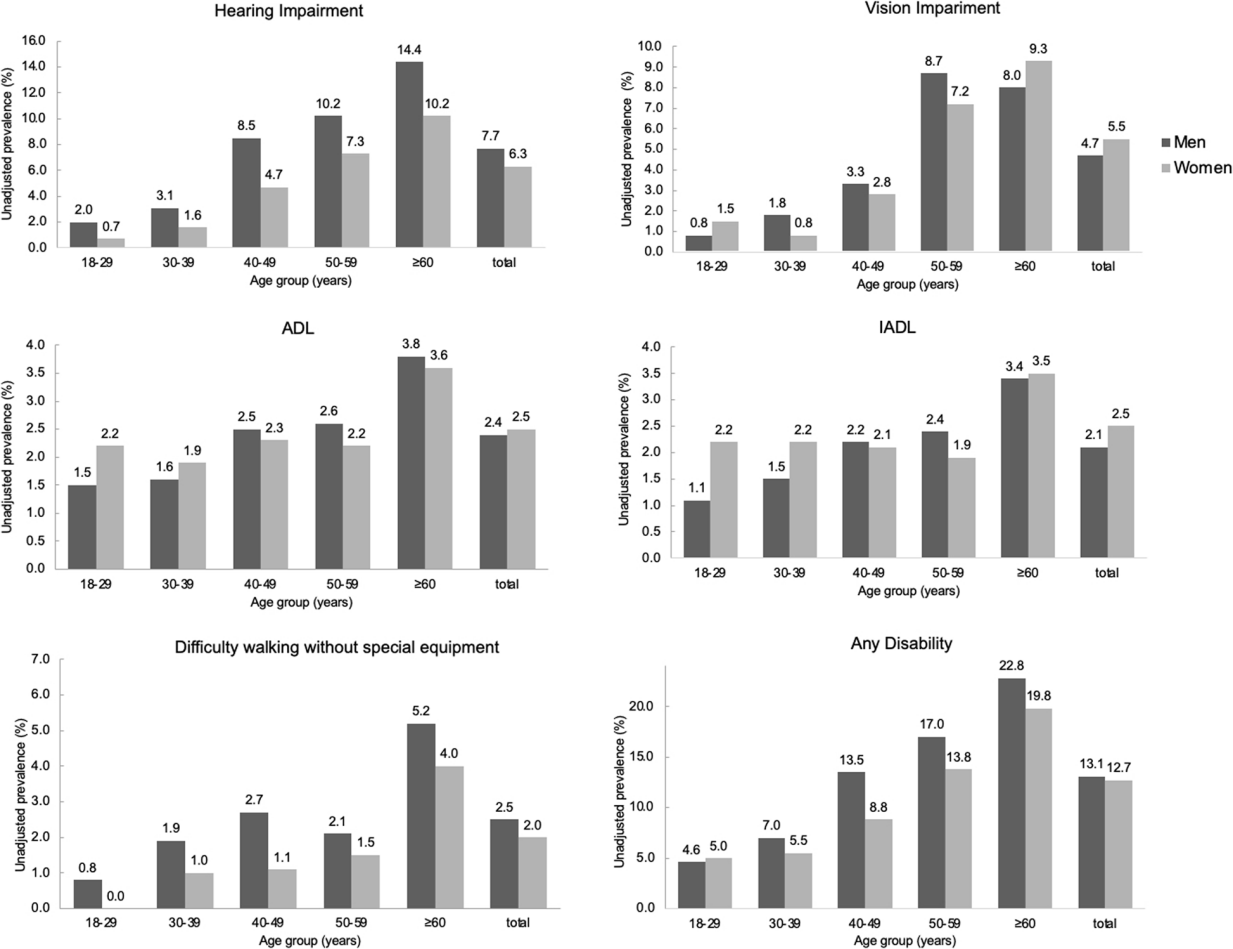
Unadjusted prevalence of hearing impairment, vision impairment, difficulty walking without special equipment, activities of daily living (ADL), instrumental activities of daily living (IADL) and any disabilities among study participants according to sex and age group in CHCN-BTH study. * Represented a *P*-value in Chi-square test was less than 0.05, and ** represented a *P*-value was less than 0.001

Figure 1 showed that the sex differences in the unadjusted prevalence of hearing impairment, vision impairment, difficulty walking without special equipment and any disabilities could be found in several age groups except 18–29 years old. Most of the prevalence were higher in men than in women. However, a significant inverse situation was observed in the vision impairment among total participants (*P* < 0.05).

**Fig. 2:**
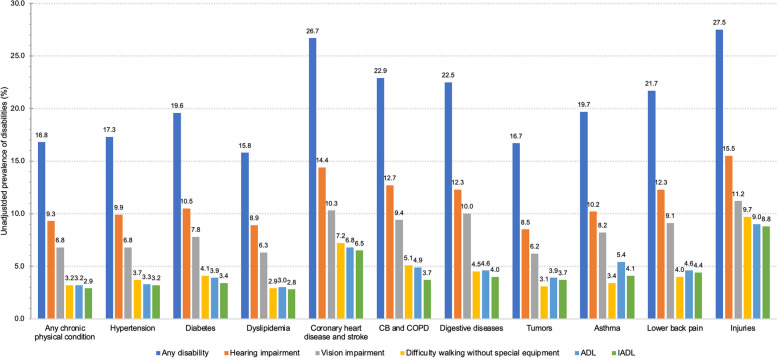
The unadjusted prevalence of hearing impairment, vision impairment, difficulty walking without special equipment, ADL, IADL and any disability in participants who had different chronic physical conditions. ADL, activities of daily living; IADL, instrumental activities of daily living

Table S1 and Fig. 2 were the unadjusted prevalence of disabilities by chronic physical conditions. We could know that participants with injuries presented the highest prevalence of hearing impairment (15.5%, 95% CI: 13.8%-17.3%), vision impairment (11.2%, 95% CI: 9.8%-12.9%), difficulty walking without special equipment (9.7%, 95% CI: 8.3%-11.3%), ADL (9.0%, 95% CI: 7.6%-10.5%), IADL (8.8%, 95% CI: 7.5%-10.3%) and any disabilities (27.5%, 95% CI: 25.4%-29.8%). Besides, participants who had coronary heart diseases and stroke, CB and COPD, digestive diseases and lower back pain also had the prevalence of any disability more than 20%.

### Multivariate and multilevel logistic regressions of disabilities with chronic physical conditions

The associations between chronic physical conditions and disabilities were shown in Table 2. In model 1, we noted that most of the chronic physical conditions were significantly associated with different types of disability after controlling for sociodemographic and behavioral variables. However, there were no significant associations between diabetes with hearing impairment (OR = 1.17, 95% CI: 0.93–1.33); hypertension (OR = 1.01, 95% CI: 0.85–1.21), diabetes (OR = 1.16, 95% CI: 0.94–1.42), tumors (OR = 1.15, 95% CI: 0.93–1.43) with vision impairment; dyslipidemia with difficulty walking without special equipment (OR = 1.16, 95% CI: 0.91–1.47), ADL (OR = 1.18, 95% CI: 0.93–1.49) and IADL (OR = 1.27, 95% CI: 0.99–1.61). Additionally, the significant associations between asthma and any types of disabilities were not observed in Model 1.

**Table 2:** Multivariate and multilevel logistic regressions of chronic physical conditions and different types of disabilities in CHCN-BTH study

	**Hearing impairment**		**Vision impairment**		**Difficulty walking without special equipment**		**ADL**		**IADL**		**Any disabilities**	
	**OR (95% CI)**	***P* value**	**OR (95% CI)**	***P* value**	**OR (95% CI)**	***P* value**	**OR (95% CI)**	***P* value**	**OR (95% CI)**	***P* value**	**OR (95% CI)**	***P* value**
**Chronic diseases**												
** Model 1**	1.47 (1.23–1.75)	< 0.001	1.50 (1.22–1.85)	< 0.001	1.98 (1.43–2.75)	< 0.001	1.84 (1.38–2.45)	< 0.001	1.84 (1.36–2.47)	< 0.001	1.57 (1.37–1.79)	< 0.001
** Model 2**	1.49 (1.43–1.54)	< 0.001	1.53 (1.24–1.88)	< 0.001	1.98 (1.45–2.72)	< 0.001	1.87 (1.43–2.44)	< 0.001	1.84 (1.41–2.41)	< 0.001	1.60*	-
**Hypertension**												
** Model 1**	1.17 (1.01–1.37)	0.042	1.01 (0.85–1.21)	0.897	1.64 (1.25–2.14)	< 0.001	1.41 (1.09–1.82)	0.009	1.67 (1.28–2.19)	< 0.001	1.19 (1.06–1.34)	0.004
** Model 2**	1.28 (1.01–1.62)	0.042	1.01 (0.83–1.22)	0.927	1.69 (1.33–2.15)	< 0.001	1.44 (1.13–1.83)	0.004	1.72 (1.39–2.14)	< 0.001	1.23 (1.03–1.47)	0.022
**Diabetes**												
** Model 1**	1.11 (0.93–1.33)	0.261	1.16 (0.94–1.42)	0.169	1.37 (1.03–1.83)	0.030	1.46 (1.09–1.96)	0.011	1.40 (1.03–1.91)	0.030	1.26 (1.10–1.45)	0.001
** Model 2**	1.18 (0.88–1.57)	0.272	1.17 (0.94–1.46)	0.156	1.39 (1.05–1.84)	0.022	1.48 (1.12–1.96)	0.005	1.41 (1.05–1.89)	0.021	1.29 (1.03–1.61)	0.022
**Dyslipidemia**												
** Model 1**	1.21 (1.05–1.40)	0.008	1.19 (1.01–1.40)	0.041	1.16 (0.91–1.47)	0.231	1.18 (0.93–1.49)	0.166	1.27 (0.99–1.61)	0.057	1.20 (1.08–1.34)	0.001
** Model 2**	1.31 (1.06–1.63)	0.014	1.20 (1.01–1.42)	0.035	1.16 (0.91–1.48)	0.237	1.18 (0.93–1.50)	0.162	1.27 (1.00–1.61)	0.048	1.22 (1.00–1.47)	0.044
**Coronary heart disease and stroke**												
** Model 1**	1.45 (1.20–1.76)	< 0.001	1.31 (1.05–1.63)	0.017	2.66 (1.99–3.54)	< 0.001	2.92 (2.17–3.92)	< 0.001	3.14 (2.32–4.26)	< 0.001	1.73 (1.49–2.02)	< 0.001
** Model 2**	1.95 (1.38–2.75)	< 0.001	1.35 (1.08–1.68)	0.008	2.85*	-	3.08*	-	3.34*	-	1.87 (1.27–2.75)	0.002
**CB and COPD**												
** Model 1**	1.47 (1.11–1.94)	0.007	1.44 (1.05–1.97)	0.024	1.68 (1.10–2.56)	0.016	1.70 (1.11–2.60)	0.014	1.38 (0.85–2.23)	0.192	1.55 (1.24–1.93)	< 0.001
** Model 2**	1.85 (1.15–2.95)	0.010	1.49 (1.08–2.04)	0.014	1.67 (1.09–2.56)	0.019	1.71 (1.13–2.59)	0.011	1.37 (0.84–2.23)	0.210	1.61 (1.09–2.38)	0.017
**Digestive diseases**												
** Model 1**	1.43 (1.16–1.76)	< 0.001	1.58 (1.26–1.99)	< 0.001	1.54 (1.10–2.15)	0.011	1.68 (1.21–2.33)	0.002	1.58 (1.12–2.24)	0.010	1.55 (1.32–1.83)	< 0.001
** Model 2**	1.77 (1.25–2.52)	0.001	1.65 (1.33–2.03)	< 0.001	1.54 (1.12–2.12)	0.008	1.70 (1.25–2.30)	< 0.001	1.58 (1.15–2.18)	0.005	1.61 (1.15–2.25)	0.005
**Tumors**												
** Model 1**	1.22 (1.01–1.48)	0.035	1.15 (0.93–1.43)	0.200	1.39 (1.03–1.90)	0.034	1.76 (1.33–2.32)	< 0.001	1.81 (1.36–2.41)	< 0.001	1.35 (1.17–1.56)	< 0.001
** Model 2**	1.28 (0.95–1.71)	0.105	0.37 (0.13–1.05)	0.061	1.34 (0.98–1.83)	0.066	1.77 (1.38–2.26)	< 0.001	1.78 (1.47–2.16)	< 0.001	1.35 (1.08–1.69)	0.008
**Asthma**												
** Model 1**	1.13 (0.65–1.95)	0.675	1.18 (0.64–2.16)	0.597	1.07 (0.43–2.65)	0.889	1.84 (0.89–3.82)	0.100	1.48 (0.65–3.34)	0.352	1.25 (0.82–1.91)	0.292
** Model 2**	1.20 (0.49–2.92)	0.687	1.22 (0.64–2.35)	0.544	1.03 (0.40–2.65)	0.947	1.92 (0.95–3.89)	0.068	1.51 (0.65–3.51)	0.334	1.31 (0.79–2.15)	0.292
**Lower back pain**												
** Model 1**	3.22 (2.79–3.71)	< 0.001	3.35 (2.83–3.96)	< 0.001	2.92 (2.29–3.71)	< 0.001	3.40 (2.69–4.30)	< 0.001	3.89 (3.03–5.00)	< 0.001	3.13 (2.80–3.49)	< 0.001
** Model 2**	3.58 (2.15–5.96)	< 0.001	7.40 (5.20–10.54)	< 0.001	3.04*	-	3.49*	-	4.02*	-	3.46 (1.70–7.03)	< 0.001
**Injuries**												
** Model 1**	2.72 (2.31–3.19)	< 0.001	2.69 (2.23–3.24)	< 0.001	8.02 (6.31–10.20)	< 0.001	6.01 (4.77–7.57)	< 0.001	6.96 (5.47–8.85)	< 0.001	2.96 (2.60–3.36)	< 0.001
** Model 2**	5.10 (3.59–7.25)	< 0.001	2.33 (1.18–4.58)	0.014	16.62 (7.23–38.20)	< 0.001	6.37*	-	7.29*	-	3.35 (2.14–5.23)	< 0.001

Model 1 was multivariate analysis adjusted for sociodemographic variables (sex, age group, BMI, the average monthly income of each family member, marital status, education, and occupation) and health behavioral variables (physical labor, physical exercise frequency, smoking and drinking). Model 2 was multilevel logistic regression model that regarded subject and regions as level 1 and level 2 based on Model 1. * Indicated that the multilevel logistic regression models were not converged, and the 95% CIs and *P* values could not be calculated. ADL, activities of daily living; IADL, instrumental activities of daily living; OR, odds ratio; CI, confidence intervals; CB, chronic bronchitis; COPD, chronic obstructive pulmonary disease; *P* < 0.05 was considered statistical significance

The results of multilevel logistic regressions (Model 2) were similar to Model 1. However, the Model 2 revealed increased ORs in the associations between lower back pain with vision impairment (Model 1: OR = 3.35, 95% CI: 2.83–3.96, AIC = 4651.5; Model 2: OR = 7.40, 95% CI: 5.20–10.54, AIC = 4634.4), as well as injuries with hearing impairment (Model 1: OR = 2.72, 95% CI: 2.31–3.19, AIC = 6059.8; Model 2: OR = 5.10, 95% CI:3.59–7.25, AIC = 6039.1) and difficulty walking without special equipment (Model 1: OR = 8.02, 95% CI: 6.31–10.20, AIC = 2414.9; Model 2: OR = 16.62, 95% CI:7.23–38.02, AIC = 2127.9), which indicating the potential effects of regions.

## Discussion

In China, plenty of disability investigations were conducted in elderly people. The current status of disability in general population was unclear. Our results provided an updated prevalence of disability in a representative cohort using a comparable assessment approach with foreign countries. In this study, 12.9% (95% CI: 12.3%-13.5%) of the respondents reported having any kind of disabilities in Beijing and Tianjin. The prevalence was similar to the national surveys of disability conducted in USA from 2009 to 2014 with the six standardized questions (11.76%-17.08%) [24], and higher than the 7% for adolescents and adults in rural China using the same assessment tool [22]. Additionally, this study revealed that the prevalence was 2.5% for ADL and 2.3% for IADL, which was consistent with a previous study using a sample of rural residents in northern China [22]. Zhang et al. [25] found that 7.9% and 18.0% of Chinese old adults had ADL and IADL disabilities, respectively. A cross-sectional study conducted in two districts of Beijing reported the prevalence of ADL was 12.1% for community elderly population [26]. Another study used six WHO SAGE countries data to report that the prevalence of ADL disability in people aged 50 years and over was 16.2% in China [15]. However, the latter three studies only included elderly Chinese population.

The discrepancy across studies might be due to the diversity of study population and investigation tools. Aging was a big challenge for the whole society. The higher proportion of unhealthy elderly people could lead to an increasing global burden and risks of age-related diseases, such as cancer, cardiovascular disease, fractures, organ failure, disability and even death [27]. Our study showed that the prevalence of disability increased with age (from 4.7% among individuals aged 18 to 29 years to 21.1% among elderly individuals aged 60 years or older), which is in line with previous studies [4, 15, 22]. Besides, the sex difference might also be a reason that related with disability. Generally, men had higher prevalence and risks of disability than women [28]. But in our study, the prevalence of vision impairment was significantly higher in women (5.5%) than men (4.7%) among total participants. The same trend could be observed in ADL and IADL but did not reach the significant level. Previous studies reported that some chronic conditions [29], mediating role of pain [30] and even depression [31]could be strong predictors for ADL disability in women, which might result in the sex-difference of the prevalence of ADL and IADL.

Except the influence of older age and sex, the Chinese criteria of disability in the previous national survey were stricter than the international community, although it was consistent with the main concepts of ICF in a way. Thus, the proportion of disabled people was relatively low, and more people with disabilities would probably be identified if measuring disability using ADL and IADL instead of the more narrowly defined impairments confirmed by a physician examination in the Second National Disability Survey in China [4]. Additionally, with the quickened urbanization and industrialization course, together with unhealthy lifestyle and other risk factors, the prevalence of chronic diseases like cancer, diabetes, COPD were significantly increasing in China [32]. WHO indicated that disability prevalence was on the rise in the years ahead, which was due to population ageing and the higher risk of disability in older people, as well as the increase in chronic health conditions such as cardiovascular disease, diabetes, and cancer [1].

WHO reported that persons with disability have higher rates of risky behaviors such as smoking and physical inactivity [1]. The current findings observed that individuals with disabilities were more likely to have reported smoking. A longitudinal study showed that cigarette smoking and obesity could increase the incidence of disability especially in younger than older adults, which might be explained by the contributing of smoking to the risk of cardiovascular diseases, respiratory diseases, and other conditions [33, 34]. Additionally, we found that people who never did physical exercise presented higher proportion of disability, which was in line with previous findings that physical inactivity was strongly associated with disability [15, 35, 36]. However, these findings need to be interpreted with caution because disability could also be the reason for physical inactivity. Further longitudinal study was needed to explore the causal associations between healthy lifestyle and disability.

Injury and lower back pain have been reported to be significant factors for disability [37, 38]. Results from this study found that 27.5% of persons with disabilities suffered an injury in the past 12 months, while only 10.9% of persons without disabilities reported any injury. In addition, compared with participants who did not have injuries, people with injuries exhibited significantly increased adjusted ORs of disabilities. Although disability especially walking difficulties might be caused by a nonfatal injury, preventing injury among persons with disabilities was of great importance as well since disability was associated with the increased risk of secondary injuries [13, 39, 40]. Studies have shown that lower back pain is a determinant of quality of life [22, 38, 41]. In addition, pain had been reported to have a strong relationship with functional limitation and disability in several studies [42–44]. Studies showed that lower back pain could be a manifestation of myopia or eyestrain, so the eye protection and eye wear would be effective measures to relief low back pain and headache, and therefore reduce the risk of long-term vision impairment [45]. Similarly, the current findings identified that 21.7% of persons with lower back pain reporting having disabilities, and individuals who had lower back pain had the highest adjusted OR of 3.46 for disability compared with those who did not have. The significant increased adjusted ORs in multilevel logistic regression models between lower back pain with vision impairments, as well as injuries with hearing impairment and walking difficulty might be due to the population difference between Tianjin and Beijing.

Our study had the strengths of well-designed protocol, larger sample size and reasonable questionnaire to investigate the disability in populations. Particularly, using the new international disability definition- The International Classification of Functioning, Disability and Health (ICF) could comprehensively explore the interactions between disability and other factors, and facilitate the comparisons across countries [8]. However, it still had several limitations. First, data for this study were derived from the baseline survey of CHCN-BTH Cohort, thus the causal associations could not be ascertained, and the possibility of reverse causality could not be ruled out as well. Further investigation using longitudinal data from the CHCN-BTH Cohort could address this problem. Second, the disability questions were self-reported, which might lead to underestimation of the true prevalence of disability. In addition, the rate of disability may be underestimated since persons who were hospitalized with serious illness during the study period could not participate in the survey and were not included. Last, the selection bias of participants could not be avoided, but all the participants were community-derived, and underwent the same inclusion and exclusion criteria. Therefore, the results of this study need to be interpreted with cautions.

## Conclusions

Findings from this study demonstrates that the many Chinese adults suffered from disabilities in Beijing and Tianjin of China. Older age, smoking, never doing exercise, obesity, and chronic physical conditions, especially injuries and lower back pain were significantly associated with disability. Sustained efforts should be made to develop specific population-based health promotion and prevention programs for disabilities in China.

### Supplementary Information

**Additional file 1: Table S1.** Unadjusted prevalence ofdisabilities by chronic physicalconditionsamong community-dwelling individuals in CHCN-BTH study.

## Data Availability

The datasets used and/or analyzed during the current study are available from the corresponding author on reasonable request.

## Abbreviations

ADL: Activities of daily living
BMI: Body mass index
CB: Chronic bronchitis
CHCN-BTH: Cohort Study on Chronic Disease of Communities Natural Population in Beijing, Tianjin and Hebei
CIs: Confidence intervals
COPD: Chronic obstructive pulmonary disease
LMICs: Low- and middle-income countries
IADL: Instrumental activities of daily living
ICF: The International Classification of Functioning, Disability and Health
NCDs: Non-communicable chronic diseases
ORs: Odds ratios
AORs: Adjusted ORs
SAGE: Study on Global AGEing and Adult Health
