# Supplementary material for: The prevalence of disability and associated factors among community adults in the baseline of CHCN-BTH Cohort Study
Source: BMC Public Health. 2023 Sep 5;23:1727. doi: 10.1186/s12889-023-15066-3 (PMC10481555; doi:10.1186/s12889-023-15066-3)
Supplement: Supplementary file 1 — Additional file 1: Table S1. Unadjusted prevalence ofdisabilities by chronic physicalconditionsamong community-dwelling individuals in CHCN-BTH study. [file 12889_2023_15066_MOESM1_ESM.docx]

**Supplementary Material**

Table S1. Unadjusted prevalence of disabilities by chronic physical conditions among community-dwelling individuals in CHCN-BTH study.

| Physical conditions | **Grouping** | **Hearing impairment** | | **Vision impairment** | | **Difficulty walking without special equipment** | | **ADL** | | **IADL** | | **Any disability** | |
| --- | --- | --- | --- | --- | --- | --- | --- | --- | --- | --- | --- | --- | --- |
|  |  | **n** | **% (95% CI)** | **n** | **% (95% CI)** | **n** | **% (95% CI)** | **n** | **% (95% CI)** | **n** | **% (95% CI)** | **n** | **% (95% CI)** |
| **Chronic diseases** | No | 213 | 3.9 (3.4-4.5) | 143 | 2.6 (2.2-3.1) | 54 | 1.0 (0.8-1.3) | 82 | 1.5 (1.2-1.9) | 75 | 1.4 (1.1-1.7) | 412 | 7.6 (6.9-8.3) |
|  | Yes | 695 | 9.3 (8.7-10.0) | 510 | 6.8 (6.3-7.4) | 241 | 3.2 (2.9-3.7) | 239 | 3.2 (2.8-3.6) | 219 | 2.9 (2.6-3.3) | 1252 | 16.8 (16.0-17.7) |
| **Hypertension** | No | 417 | 5.3 (4.8-5.8) | 312 | 4.0 (3.5-4.4) | 112 | 1.4 (1.2-1.7) | 157 | 2.0 (1.7-2.3) | 134 | 1.7 (1.4-2.0) | 801 | 10.1 (9.5-10.8) |
|  | Yes | 491 | 9.9 (9.1-10.7) | 341 | 6.8 (6.2-7.6) | 183 | 3.7 (3.2-4.2) | 164 | 3.3 (2.8-3.8) | 160 | 3.2 (2.8-3.7) | 863 | 17.3 (16.3-18.4) |
| **Diabetes** | No | 733 | 6.5 (6.1-7.0) | 524 | 4.7 (4.3-5.1) | 227 | 2.0 (1.8-2.3) | 257 | 2.3 (2.0-2.6) | 237 | 2.1 (1.9-2.4) | 1339 | 11.9 (11.4-12.6) |
|  | Yes | 175 | 10.5 (9.1-12.1) | 129 | 7.8 (6.5-9.1) | 68 | 4.1 (3.2-5.1) | 64 | 3.9 (3.0-4.9) | 57 | 3.4 (2.6-4.4) | 325 | 19.6 (17.7-21.5) |
| **Dyslipidemia** | No | 463 | 5.9 (5.4-6.4) | 335 | 4.3 (3.8-4.7) | 147 | 1.9 (1.6-2.2) | 172 | 2.2 (1.9-2.5) | 153 | 1.9 (1.7-2.3) | 870 | 11.1 (10.4-11.8) |
|  | Yes | 445 | 8.9 (8.1-9.7) | 318 | 6.3 (5.7-7.0) | 148 | 2.9 (2.5-3.4) | 149 | 3.0 (2.5-3.5) | 141 | 2.8 (2.4-3.3) | 794 | 15.8 (14.8-16.8) |
| **Coronary heart disease and stroke** | No | 744 | 6.3 (5.9-6.8) | 536 | 4.6 (4.2-5.0) | 213 | 1.8 (1.6-2.1) | 244 | 2.1 (1.8-2.3) | 220 | 1.9 (1.7-2.3) | 1360 | 11.6 (11.0-12.2) |
|  | Yes | 164 | 14.4 (12.5-16.6) | 117 | 10.3 (8.6-12.2) | 82 | 7.2 (5.8-8.8) | 77 | 6.8 (5.4-8.3) | 74 | 6.5 (5.2-8.1) | 304 | 26.7 (24.2-29.4) |
| **CB and COPD** | No | 843 | 6.8 (6.4-7.3) | 605 | 4.9 (4.5-5.3) | 269 | 2.2 (1.9-2.4) | 296 | 2.4 (2.1-2.7) | 275 | 2.2 (2.0-2.5) | 1547 | 12.5 (11.9-13.1) |
|  | Yes | 65 | 12.7 (10.0-15.8) | 48 | 9.4 (7.1-12.2) | 26 | 5.1 (3.4-7.3) | 25 | 4.9 (3.3-7.0) | 19 | 3.7 (2.3-5.6) | 117 | 22.9 (19.4-26.7) |
| **Digestive diseases** | No | 784 | 6.6 (6.2-7.1) | 552 | 4.7 (4.3-5.0) | 250 | 2.1 (1.9-2.4) | 275 | 2.3 (2.0-2.5) | 254 | 2.1 (1.9-2.4) | 1437 | 12.1 (11.5-12.7) |
|  | Yes | 124 | 12.3 (10.4-14.4) | 101 | 10 (8.3-12.0) | 45 | 4.5 (3.3-5.9) | 46 | 4.6 (3.4-6.0) | 40 | 4.0 (2.9-5.3) | 227 | 22.5 (20.0-25.2) |
| **Tumors** | No | 751 | 6.8 (6.4-7.3) | 537 | 4.9 (4.5-5.3) | 238 | 2.2 (1.9-2.4) | 248 | 2.3 (2.0-2.5) | 225 | 2.0 (1.8-2.3) | 1353 | 12.3 (11.7-12.9) |
|  | Yes | 157 | 8.5 (7.3-9.8) | 116 | 6.2 (5.2-7.4) | 57 | 3.1 (2.4-3.9) | 73 | 3.9 (3.1-4.9) | 69 | 3.7 (2.9-4.6) | 311 | 16.7 (15.1-18.5) |
| **Asthma** | No | 893 | 7.0 (6.6-7.5) | 641 | 5.0 (4.7-5.4) | 290 | 2.3 (2.0-2.5) | 313 | 2.5 (2.2-2.7) | 288 | 2.3 (2.0-2.5) | 1635 | 12.8 (12.3-13.4) |
|  | Yes | 15 | 10.2 (6.1-15.9) | 12 | 8.2 (4.5-13.4) | 5 | 3.4 (1.3-7.3) | 8 | 5.4 (2.6-10.0) | 6 | 4.1 (1.7-8.2) | 29 | 19.7 (13.9-26.7) |
| **Lower back pain** | No | 362 | 4.3 (3.9-4.8) | 247 | 2.9 (2.6-3.3) | 116 | 1.4 (1.1-1.6) | 114 | 1.4 (1.1-1.6) | 96 | 1.1 (0.9-1.4) | 697 | 8.3 (7.7-8.9) |
|  | Yes | 546 | 12.3 (11.3-13.2) | 406 | 9.1 (8.3-10.0) | 179 | 4.0 (3.5-4.6) | 207 | 4.6 (4.105.3) | 198 | 4.4 (3.9-5.1) | 967 | 21.7 (20.5-22.9) |
| **Injuries** | No | 663 | 5.9 (5.5-6.3) | 475 | 4.2 (3.8-4.6) | 141 | 1.2 (1.1-1.5) | 179 | 1.6 (1.4-1.8) | 155 | 1.4 (1.2-1.6) | 1228 | 10.9 (10.3-11.5) |
|  | Yes | 245 | 15.5 (13.8-17.3) | 178 | 11.2 (9.8-12.9) | 154 | 9.7 (8.3-11.3) | 142 | 9.0 (7.6-10.5) | 139 | 8.8 (7.5-10.3) | 436 | 27.5 (25.4-29.8) |

ADL, activities of daily living; IADL, instrumental activities of daily living; CB, chronic bronchitis; COPD, chronic obstructive pulmonary disease; CI, confidence intervals.
